# Paraplegia due to Thoracic Mobile Schwannoma after Myelography

**DOI:** 10.1155/2020/6709819

**Published:** 2020-09-14

**Authors:** Akira Honda, Yoichi Iizuka, Tokue Mieda, Hiroyuki Sonoda, Sho Ishiwata, Yohei Kakuta, Daisuke Tsunoda, Eiji Takasawa, Tsuyoshi Tajika, Hiromi Koshi, Hirotaka Chikuda

**Affiliations:** ^1^Department of Orthopaedic Surgery, Gunma University Graduate School of Medicine, 3-39-22, Showa, Maebashi, Gunma 371-8511, Japan; ^2^Clinical Department of Pathology, Gunma University Hospital, 3-39-22, Showa, Maebashi, Gunma 371-8511, Japan

## Abstract

**Introduction:**

Spinal mobile tumors are very rare. We herein report a case of paraplegia caused by migration and incarceration of thoracic mobile schwannoma after myelography. *Case Presentation*. A 25-year-old man who had weakness and numbness in both his legs also had pain radiating to the back that was induced by back flexion or extension and jumping. Magnetic resonance imaging (MRI) showed an intradural extramedullary lesion at the T10 and T11 levels. Myelography was performed but discontinued due to his back and lower limb pain. Computed tomography after myelography revealed a rostrally migrated intradural mass with a discrepancy in the exact location in comparison to the MRI findings. He underwent a second lumbar puncture and drained the cerebrospinal fluid (CSF) to aid the spinal cord, because the symptoms gradually worsened and led to paraplegia. After the drainage of the CSF, his symptoms were immediately resolved. The day after myelography, he underwent complete resection of the tumor with the diagnosis of schwannoma. One year after the surgery, he had been working despite having hyperreflexia in his lower limbs with no weakness or sensory disturbance.

**Conclusion:**

Severe neurological deficits associated with spinal cord damage can occur due to migration of mobile tumors.

## 1. Introduction

Spinal mobile tumors are very rare. Migration of mobile tumors can occur due to various causes, including postural changes, trauma and intrathecal injection of contrast medium used in myelography [1–5]. Although previous case reports have mainly discussed ways to avoid a misdiagnosis of the level of the spinal mobile tumor [1–4], there have been none regarding the adverse events due to migration of the mobile tumor caused by myelography.

We herein report a case of paraplegia caused by migration and incarceration of thoracic mobile schwannoma after myelography.

## 2. Case Presentation

A 25-year-old man presented with a 1-month history of weakness and numbness in both his legs. At his first visit, neurological examination revealed normal muscle strength with a muscle stretch reflex indicating hyperreflexia in the lower limbs. In addition, pain radiating to the back was induced by back flexion or extension and jumping. He was ambulatory, although a spastic gait was evident.

**Figure 1:**
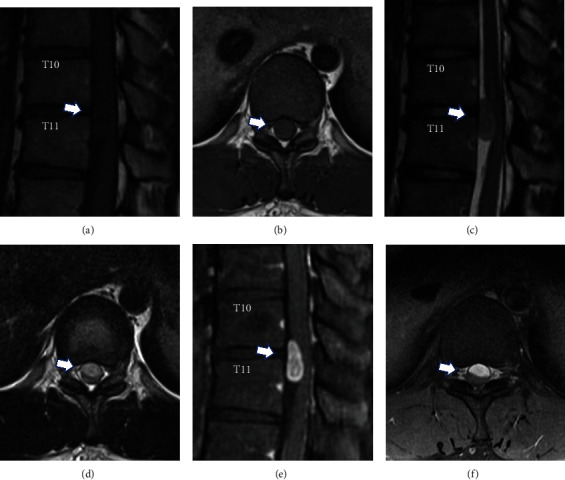
Magnetic resonance imaging of a 25-year-old man revealed an intradural extramedullary lesion at the T10 and T11 levels. The lesion shows hypointense in T1-weighted (a, b), isointense in T2-weighted (c, d) and heterogeneous in gadolinium enhancement (e, f) sagittal and axial magnetic resonance imaging of the thoracic spine. Arrows indicate the location of the lesion.

**Figure 2:**
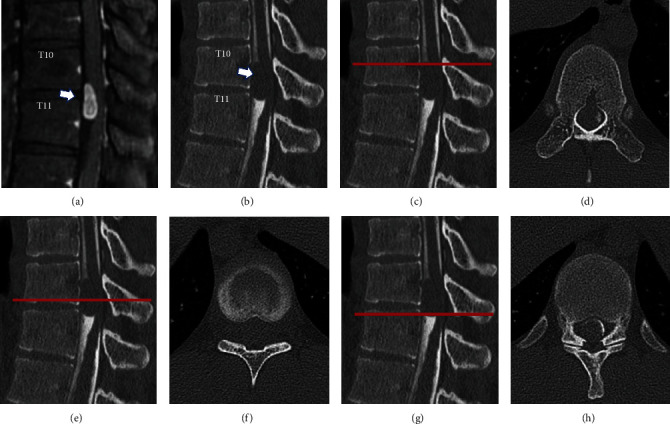
Magnetic resonance imaging before myelography (a) and computed tomography after the myelography (b) shows the intradural mass rostrally migrated with approximately 10 mm discrepancy in the exact location compared to magnetic resonance imaging findings. Arrows indicate the location of the lesion. Each level of axial image on computed tomography shows the proximal (c, d), middle (e, f), and distal (g, h) levels of the tumor.

Magnetic resonance imaging (MRI) showed an intradural extramedullary lesion with hypointense changes on T1 and isointense changes on T2 as well as heterogeneous gadolinium enhancement at the T10 and T11 levels (Figure 1). Although MRI findings appeared circumscribed, the mass was large and was located on the ventrolateral side. An accurate preoperative diagnosis was considered important; thus, we performed myelography to obtain more detailed information. Injection of iohexol was performed following puncture at the L4 and L5 levels, and 1 ml of cerebrospinal fluid (CSF) was collected for a CSF analysis. He reported back and lower limb pain after the injection of 2 ml of iohexol. As he reported severe pain just after the injection of 5 ml of iohexol, 3 ml of CSF was drained, and the pain improved immediately. Computed tomography (CT) after myelography revealed a rostrally migrated intradural mass with an approximately 10 mm discrepancy in the exact location compared to MRI findings (Figure 2). After myelography, weakness and decreased sensation in the lower limbs gradually developed, resulting in paraplegia with a muscle strength of grade 0/5. A second lumbar puncture was performed, and 10 ml of CSF was drained. After CSF drainage, the symptoms—including weakness and sensory disturbance—immediately improved.

**Figure 3:**
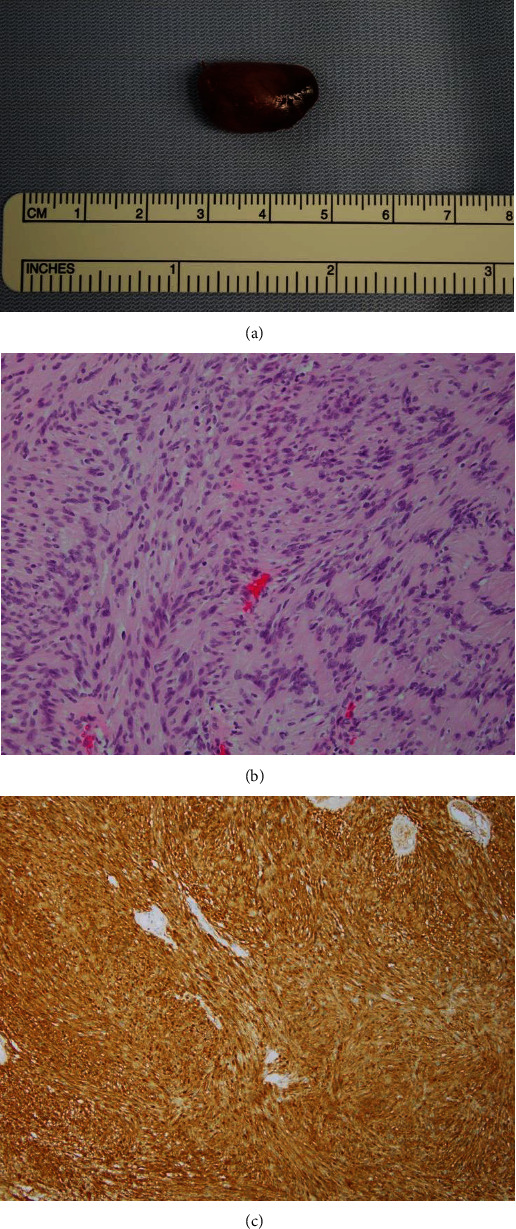
A well-capsulated, dark-reddish tumor was resected in an en bloc fashion (a). Histological images (b) showing Antoni A-positive areas composed of spindle cells with nuclear palisading (hematoxylin and eosin stain). No marked nuclear atypia, mitotic figures, or necrosis was seen. S-100 protein was strongly expressed (c).

**Figure 4:**
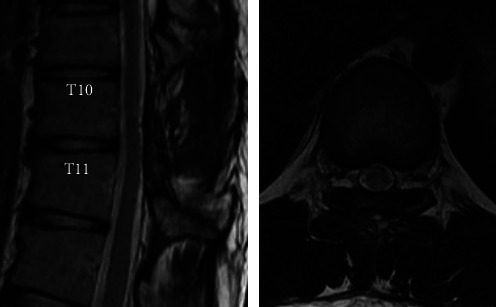
Magnetic resonance imaging one year after operation shows a completely resected and well-decompressed spinal cord.

Surgical treatment the day after myelography achieved complete resection of the intradural extramedullary tumor, and a histological examination confirmed the diagnosis of schwannoma (Figure 3). At 12 months after surgery (Figure 4), he was working as a physical education teacher despite having hyperreflexia in his lower limbs.

## 3. Discussion

Spinal mobile tumor was first described in 1963 by Wortzman and Botterell, and it was an ependymoma of the filum terminale with unusual laxity [6]. Although some case reports of spinal mobile tumors have been published, most of those tumors were the cauda equina tumors, and thoracic mobile schwannoma has been proven extremely rare [7–13].

Intrathecal pressure-associated or position-associated symptoms, including pain aggravated by coughing or sneezing or a supine position, have been described in some previous studies [4, 14]. In the present case, radiating pain was induced by back extension/flexion and jumping and was considered associated with intrathecal pressure or postural change. Therefore, provocative maneuvers, such as back extension/flexion and jumping, may be useful for assessing the mobility of spinal tumors.

The migration of spinal tumors is recognized as a possible event that is caused by the intrathecal injection of contrast medium during myelography [1, 4, 5]. Tavy et al. [4] reported that preoperative myelography revealed a schwannoma of the cauda equina at T12-L3 despite the fact that the tumor had been located at the upper margin of L2. Varughese and Mazagri [1] also reported that CT images following myelography showed a migrated intradural schwannoma at L3 that had been observed at L4 just after myelography. Although migration may be caused by myelography, there have been no reports regarding paraplegia after the induction of tumor migration by myelography. In the present case, the tumor was considered to have migrated rostrally after the injection of contrast medium because the symptom did not occur during the puncture procedure. This suggests that the increase in intrathecal pressure below the tumor due to injection caused the migration rather than a CSF pressure gradient related to the puncture itself.

The preoperative diagnosis of spinal cord tumors remains difficult, and the surgical treatment of spinal cord lesions is still challenging. Spinal schwannomas mostly originate from the dorsal root in the cervical or lumbar and sacral segments and rarely occur on the ventrolateral side of the spinal cord, whereas meningiomas are most commonly located lateral or ventrolateral to the spinal cord in the thoracic spine [15, 16]. In the present case, the tumor was large and was located ventrolateral to the spinal cord in the thoracic spine. An accurate preoperative diagnosis is important, because the surgical strategy usually differs between schwannomas and meningiomas. In patients with a large mass and severe spinal compression, CT myelography was reported to be more accurate for the delineation of the spinal cord and tumor mass than MRI [17]. Thus, we performed myelography to obtain more detailed information. Spinal cord damage occurred as a consequence after myelography and was considered to be due to incarceration of the thoracic schwannoma associated with increased intrathecal pressure below the tumor. Although we considered emergency surgery, we first performed a second puncture to manage the spinal cord based on evidence of successful drainage in the first procedure, and of note, the neurological condition immediately recovered. Fortunately, the severe neurological deficit associated with the spinal cord damage was resolved immediately by CSF drainage; however, it might be better to avoid myelography in patients with symptoms suggestive of mobile spinal tumors, especially in those with tumors at the spinal cord level.

## 4. Conclusion

This is the first reported case of paraplegia caused by migration and incarceration of thoracic mobile schwannoma after myelography. The potential for severe neurological deficits after myelopathy should be taken into account in patients with spinal tumors, particularly those at the spinal cord level.

## Additional Points

*Copyright Form*. If and when the manuscript is accepted for publication, the authors agree to automatic transfer of the copyright to the publisher.
